# A MALDI-TOF MS database with broad genus coverage for species-level identification of *Brucella*

**DOI:** 10.1371/journal.pntd.0006874

**Published:** 2018-10-18

**Authors:** Jennifer Mesureur, Sandrine Arend, Béatrice Cellière, Priscillia Courault, Pierre-Jean Cotte-Pattat, Heather Totty, Parampal Deol, Virginie Mick, Victoria Girard, Joanne Touchberry, Vanessa Burrowes, Jean-Philippe Lavigne, David O’Callaghan, Valérie Monnin, Anne Keriel

**Affiliations:** 1 VBMI, Inserm, Université de Montpellier, Nimes, France; 2 Centre National de Référence des *Brucella*, CHU Caremeau, Nimes, France; 3 bioMérieux France, La Balme-les-Grottes, France; 4 bioMérieux Inc, Durham, North Carolina, United States of America; 5 Anses/Paris-Est University, EU/OIE/FAO & National Reference Laboratory for Brucellosis, Animal Health Laboratory, Maisons-Alfort, France; 6 North Carolina State Laboratory of Public Health, Raleigh, North Carolina, United States of America; 7 Service de Microbiologie, CHU Caremeau, Nimes, France; University of Minnesota, UNITED STATES

## Abstract

*Brucella* are highly infectious bacterial pathogens responsible for a severely debilitating zoonosis called brucellosis. Half of the human population worldwide is considered to live at risk of exposure, mostly in the poorest rural areas of the world. Prompt diagnosis of brucellosis is essential to prevent complications and to control epidemiology outbreaks, but identification of *Brucella* isolates may be hampered by the lack of rapid and cost-effective methods. Nowadays, many clinical microbiology laboratories use Matrix-Assisted Laser Desorption Ionization–Time Of Flight mass spectrometry (MALDI-TOF MS) for routine identification. However, lack of reference spectra in the currently commercialized databases does not allow the identification of *Brucella* isolates. In this work, we constructed a *Brucella* MALDI-TOF MS reference database using VITEK MS. We generated 590 spectra from 84 different strains (including rare or atypical isolates) to cover this bacterial genus. We then applied a novel biomathematical approach to discriminate different species. This allowed accurate identification of *Brucella* isolates at the genus level with no misidentifications, in particular as the closely related and less pathogenic *Ochrobactrum* genus. The main zoonotic species (*B*. *melitensis*, *B*. *abortus* and *B*. *suis*) could also be identified at the species level with an accuracy of 100%, 92.9% and 100%, respectively. This MALDI-TOF reference database will be the first *Brucella* database validated for diagnostic and accessible to all VITEK MS users in routine. This will improve the diagnosis and control of brucellosis by allowing a rapid identification of these pathogens.

## Author summary

*Brucella* are bacteria that mainly infect animals. They can also be transmitted to humans and cause a serious disease called brucellosis. Half the world's population is considered exposed, especially in the poorest rural areas. Experts agree that prompt identification of *Brucella* isolates is essential to provide appropriate treatment to patients and to control epidemiological outbreaks. Mis-identification of these highly infectious pathogens may lead to delays in diagnosis, but also to increased risks of accidental exposure for laboratory workers. MALDI-TOF mass spectrometry is now the first line of bacterial identification in many routine diagnostic laboratories. However, not all clinical mass spectrometers can identify *Brucella*. In this work, we updated a database with *Brucella* spectra to improve the performance of MALDI-TOF mass spectrometers. These instruments will now be able to identify accurately *Brucella* isolates. This will greatly improve the diagnosis of brucellosis.

## Introduction

*Brucella* are important pathogens in medical and veterinary context. These Gram-negative bacteria can be transmitted from their animal reservoir to humans, usually by ingestion of contaminated milk products or direct contact, causing brucellosis. This zoonosis causes a severely debilitating illness characterized by intermittent fever, chills, sweats, weakness, myalgia, osteoarticular or obstetrical complications and endocarditis.

This disease is largely unreported and the true incidence of human brucellosis is thus unknown [1]. According to the World Health Organization (WHO), half a million new cases are reported each year, most of them in the poorest rural areas of the world [2]. Indeed, while the disease has been successfully prevented in most industrialized countries, it remains a significant burden in the Mediterranean region, all over Asia, sub-Saharan Africa, and certain areas in Latin America. Approximately half of the human population worldwide is considered to live at risk of exposure [3]. Moreover, due to the low dose required to cause infection (10–100 colony-forming units) and the potential for aerosol dissemination, *Brucella* was considered a potential bioterrorism agent early in the 20th century [1] and its possession and use is still strictly regulated in many countries.

Currently, the *Brucella* genus consists of eleven recognized species plus several isolates that have not yet been officially designated. The major zoonotic species are *B*. *melitensis*, *B*. *abortus* and *B*. *suis* which are subdivided into biovars by a set of phenotypic characteristics including lipopolysaccharide (LPS) epitopes, phage sensitivity, dye sensitivity and a battery of biochemical tests. These three species are also the most common in domestic livestock. *B*. *melitensis* is responsible for the majority of human cases in the Mediterranean basin, the Arab peninsula, Latin America countries and Asia, while *B*. *abortus* is more prevalent in the United States, Northern Europe and Africa [4]. *B*. *suis* and *B*. *canis* infections are more sporadic in humans. Very rare human infections have also been reported with *B*. *inopinata* [5,6], *B*. *ceti* [7,8] and *B*. *neotomae* [9,10].

Clinical microbiology laboratories play a key role in the diagnosis and management of human brucellosis and should be able to provide a rapid and exact identification of *Brucella* spp. Currently, the most suitable tool for identification of bacteria is Matrix Assisted Laser Desorption/Ionization-Time of Flight Mass Spectrometry (MALDI-TOF MS). This method provides rapid, sensitive and cost-effective identification and is currently replacing phenotypic microbial identification. Its accuracy however largely depends on the coverage of the database of the commercially available MALDI-TOF MS systems. With regards to *Brucella*, identification was not possible because this genus was not represented in the databases of the two main MALDI-TOF MS system manufacturers (*i*.*e*. bioMérieux and Bruker) [11–13]. Only the Bruker Security Relevant (SR) database, or custom databases developed in some laboratories, can identify these highly pathogenic bacteria, but access to these databases is not possible in some countries due to export restriction regulations [13–15]. Moreover, only *B*. *melitensis* is included in the SR database.

## Material and methods

### Bacterial strains

The bacterial strains used for the construction of the database are listed in Tables 1 and S1. Each of these strains was cultivated on several different media (S1 Table). The bacterial isolates used for the external evaluation and their culture conditions are listed in Tables 2 and S2. All strains used in this study were previously characterized using an established workflow (phenotypic assays, Multiple-Locus Variable number tandem repeat Analysis -or MLVA-, whole-genome sequencing) [16].

**Table 1:** *Brucella* strains and isolates retained to generate the MALDI-TOF MS database. Strains highlighted in grey are reference or type strains.

Name	Species (biovar)	Origin
16M	*B*. *melitensis* (1)	Veterinary isolate (goat)
95-5009-1	*B*. *melitensis* (1)	Veterinary isolate (sheep)
95-2426-961	*B*. *melitensis* (1)	Veterinary isolate (sheep)
13-2582-4590	*B*. *melitensis* (1)	Clinical isolate
05–0737	*B*. *melitensis* (1)	Clinical isolate (blood)
08-2437-5214	*B*. *melitensis* (1)	Veterinary isolate (goat)
63/9	*B*. *melitensis* (2)	Clinical isolate (blood)
11-939-2005	*B*. *melitensis* (2)	Clinical isolate (blood)
04–1553	*B*. *melitensis* (2)	Clinical isolate (blood)
Ether	*B*. *melitensis* (3)	Clinical isolate
11-441-982	*B*. *melitensis* (3)	Clinical isolate (blood)
10–394	*B*. *melitensis* (3)	Clinical isolate (blood)
07-1184-2852	*B*. *melitensis* (3)	Clinical isolate (blood)
02–1213	*B*. *melitensis* (3)	Veterinary isolate (cow)
05–0682	*B*. *melitensis* (3)	Clinical isolate (blood)
11-2159-4003	*B*. *melitensis* (3)	Clinical isolate (blood)
B115	*B*. *melitensis*	Veterinary isolate (goat)
BT020216	*B*. *melitensis*	Clinical isolate
BT071315-0001	*B*. *melitensis*	Clinical isolate
BT072914	*B*. *melitensis*	Clinical isolate
BT1202150001	*B*. *melitensis*	Clinical isolate
544	*B*. *abortus* (1)	Veterinary isolate (cow)
01–673	*B*. *abortus* (1)	Veterinary isolate (cow)
03-2770-3	*B*. *abortus* (1)	Veterinary isolate (cow)
05-147-200	*B*. *abortus* (1)	Veterinary isolate (cow)
93–12101	*B*. *abortus* (1)	Clinical isolate (blood)
2000031295	*B*. *abortus* (1)	Clinical isolate
86/8/59	*B*. *abortus* (2)	Veterinary isolate (cow)
03-2770-11	*B*. *abortus* (2)	Veterinary isolate (cow)
92–601	*B*. *abortus* (2)	Veterinary isolate (cow)
Tulya	*B*. *abortus* (3)	Clinical isolate
12–1745	*B*. *abortus* (3)	Veterinary isolate (cow)
03–2055	*B*. *abortus* (3)	Veterinary isolate (sheep)
99–4566	*B*. *abortus* (3)	Veterinary isolate (cow)
03–4278	*B*. *abortus* (3)	Clinical isolate (blood)
92-7369-2	*B*. *abortus* (3)	Veterinary isolate (cow)
292	*B*. *abortus* (4)	Veterinary isolate (cow)
99–9473	*B*. *abortus* (4)	Veterinary isolate (cow)
B3196	*B*. *abortus* (5)	Veterinary isolate (cow)
870	*B*. *abortus* (6)	Veterinary isolate (cow)
C68	*B*. *abortus* (9)	Veterinary isolate (cow)
1330	*B*. *suis*	Veterinary isolate (pig)
13-896-1815	*B*. *suis* (1)	Clinical isolate
12-2826-5972	*B*. *suis* (1)	Clinical isolate
04-1361Djakovo-1	*B*. *suis* (1)	Veterinary isolate (boar)
11-2920-5143	*B*. *suis* (1)	Clinical isolate (blood)
05–4266	*B*. *suis* (1)	Clinical isolate (blood)
Thompsen	*B*. *suis* (2)	Veterinary isolate (hare)
12-4327-8815	*B*. *suis* (2)	Veterinary isolate (hare)
12-2885-6046	*B*. *suis* (2)	Clinical isolate
11-3301-6219	*B*. *suis* (2)	Veterinary isolate (pig)
11-2942-5156	*B*. *suis* (2)	Veterinary isolate (boar)
11-028-111	*B*. *suis* (2)	Veterinary isolate (pig)
09-372-779	*B*. *suis* (2)	Veterinary isolate (sheep)
05–3495	*B*. *suis* (2)	Clinical isolate (hip prosthesis)
00–4898	*B*. *suis* (2)	Veterinary isolate (cow)
686	*B*. *suis* (3)	Veterinary isolate (reindeer)
40	*B*. *suis* (4)	Veterinary isolate (reindeer)
513	*B*. *suis* (5)	Veterinary isolate (rodent)
03-2770-12	*B*. *canis*	Veterinary isolate (dog)
04-2330-1	*B*. *canis*	Veterinary isolate (dog)
09-369-776(2)	*B*. *canis*	Veterinary isolate (dog)
11-1961-3694(1)	*B*. *canis*	Veterinary isolate (dog)
08-1276-2270	*B*. *canis*	Clinical isolate (blood)
63/290	*B*. *ovis*	Veterinary isolate (sheep)
12-1497-b	*B*. *ovis*	Veterinary isolate (sheep)
11-868-1991	*B*. *ovis*	Veterinary isolate (sheep)
12-3480-79	*B*. *ovis*	Veterinary isolate (sheep)
B1/94	*B*. *ceti*	Veterinary isolate (porpoise)
34/94	*B*. *ceti*	Veterinary isolate (porpoise)
97/0776	*B*. *ceti*	Veterinary isolate (dolphin)
B202R	*B*. *ceti*	Veterinary isolate (whale)
47/94	*B*. *ceti*	Veterinary isolate (dolphin)
B14/94	*B*. *ceti*	Veterinary isolate (dolphin)
98/230	*B*. *ceti*	Veterinary isolate (dolphin) [17]
5/95	*B*. *ceti*	Veterinary isolate (dolphin)
B2/94	*B*. *pinnipedialis*	Veterinary isolate (seal)
39/94	*B*. *pinnipedialis*	Veterinary isolate (seal)
55/94	*B*. *pinnipedialis*	Veterinary isolate (otter)
61/94	*B*. *pinnipedialis*	Veterinary isolate (seal)
BO1	*B*. *inopinata*	Clinical isolate (breast implant) [5]
BO2	*B*. *inopinata*-like	Clinical isolate (lung) [6]
F8/08-60	*B*. *papionis*	Veterinary isolate (baboon) [18]
F8/08-61	*B*. *papionis*	Veterinary isolate (baboon) [18]

**Table 2:** Bacterial strains and isolates used for external validation of the database.

Name	Species (biovar)	Description
2308	*B*. *abortus* (1)	Veterinary isolate (cow)
S19	*B*. *abortus* (1)	Vaccine strain (spontaneous attenuation)
RB51	*B*. *abortus* (1)	Vaccine strain (rough mutant)
97-4775-11	*B*. *abortus* (1)	Veterinary isolate (cow)
75–17	*B*. *abortus* (3)	Veterinary isolate (cow)
83–227	*B*. *abortus* (3)	Veterinary isolate (cow)
83–233	*B*. *abortus* (3)	Veterinary isolate (cow)
79–153	*B*. *abortus* (3)	Veterinary isolate (cow)
82–41	*B*. *abortus* (3)	Veterinary isolate (cow)
99-9971-135	*B*. *abortus* (7)	Veterinary isolate (cow) [21]
03-4923-239-D	*B*. *abortus* (7)	Veterinary isolate (cow) [21]
77–9	*B*. *abortus* (9)	Veterinary isolate (cow)
80–133	*B*. *abortus* (9)	Veterinary isolate (dog)
Mex 51	*B*. *canis*	Veterinary isolate (dog)
36/94	*B*. *ceti*	Veterinary isolate (porpoise) [22]
F5/99	*B*. *ceti*	Veterinary isolate (dolphin) [23]
UK3/05	*B*. *ceti*	Veterinary isolate (dolphin) [24]
75–3	*B*. *melitensis* (1)	Clinical isolate
78–158	*B*. *melitensis* (1)	Veterinary isolate (sheep)
88–44	*B*. *melitensis* (1)	Clinical isolate
1109	*B*. *melitensis* (1)	Clinical isolate
Rev1	*B*. *melitensis* (1)	Vaccine strain
16M+GFP	*B*. *melitensis* (1)	Fluorescent 16M strain
72–59	*B*. *melitensis* (3)	Veterinary isolate (goat)
77–47	*B*. *melitensis* (3)	Clinical isolate
78–13	*B*. *melitensis* (3)	Veterinary isolate (goat)
81–44	*B*. *melitensis* (3)	
81–140	*B*. *melitensis* (3)	Clinical isolate
82–73	*B*. *melitensis* (3)	Clinical isolate
82–87	*B*. *melitensis* (3)	Clinical isolate
90–129	*B*. *melitensis* (3)	Veterinary isolate (cow)
91–244	*B*. *melitensis* (3)	Veterinary isolate (sheep)
79–185	*B*. *melitensis* (3)	Clinical isolate
CCM4915	*B*. *microti*	Type strain (BCCN 07–01) [25]
5K33	*B*. *neotomae*	Type strain (ATCC 23459) [26]
76250	*B*. *ovis*	
91268	*B*. *ovis*	
91212	*B*. *ovis*	
56/94	*B*. *pinnipedialis*	Veterinary isolate (seal) [22]
96/408	*B*. *pinnipedialis*	Veterinary isolate (seal) [23]
UK9/99	*B*. *pinnipedialis*	Veterinary isolate (seal) [24]
04-1361Sisak-4	*B*. *suis* (1)	Veterinary isolate (boar)
15/95	*Brucella sp*.	Veterinary isolate (seal)
49/94	*Brucella sp*.	Veterinary isolate (dolphin)
NF2637	*Brucella sp*.	Veterinary isolate (rodents) [27]
NF2653	*Brucella sp*.	Veterinary isolate (rodents) [27]
02/611	*Brucella sp*.	Clinical isolate (osteomyelitis) [7].
B13-0095	*Brucella sp*.	Veterinary isolate (frog) [28]
ATCC48188	*Ochrobactrum anthropi*	Reference strain
LMG3301	*Ochrobactrum intermedium*	Type strain

### MALDI-TOF MS samples

Samples used to build the spectra database were prepared according to a previously established inactivation protocol [19] consisting in resuspending two full loops of bacteria (*i*.*e*. multiple colonies) in 200 μL of solvent mix, vortexing (10 sec), centrifuging (10,000 g, 2 min) at room temperature, removing 190 μL and resuspending in the 10 μL of solvent left in the tube. For the external evaluation study, this protocol was simplified by suspending only one loop of bacteria in 100 μL of solvent mix, vortexing (10 sec) and incubating at room temperature (20–25°C, 3 minutes). Bacteria were efficiently inactivated by this method and the biomass concentration of the samples allowed identification by MALDI-TOF MS, demonstrating that the centrifugation step in the original protocol was not required.

### MALDI-TOF MS analysis

One μL of each sample was applied to a single well of a disposable, barcode-labeled target slide (VITEK MS-DS, bioMérieux), overlaid with 1 μL of a saturated solution of alpha-cyano-4-hydroxycinnamic acid matrix in 50% acetonitrile and 2.5% trifluoroacetic acid (VITEK MSCHCA, bioMérieux) then air dried. For the database construction, several independent measurements were recorded for each strain (see S1 Table for the different culture conditions).

For instrument calibration, an *Escherichia coli* reference strain (ATCC 8739) was directly transferred to designated spots on the target slide using the procedure recommended by the manufacturer.

Mass spectra were acquired using a VITEK MS Plus (bioMérieux, Marcy l’Etoile) and the Launchpad v2.8 software program (Kratos, Shimadzu group Compagny, Manchester, UK). Dendrograms showing taxonomic relationships between strains were constructed using the SARAMIS software (bioMérieux, Marcy l’Etoile, France).

### Construction and optimization of the database

The database was built as previously described [20]. Briefly, peak lists were binned by assigning each peak within the mass range of 3.000–17.000 Da to one of 1,300 bins. A predictive model was then established for each species using the Advanced Spectra Classifier (ASC) algorithm developed by bioMérieux (La Balme les Grottes, France). The outcome of this procedure provided an assignment of a dimensionless weight for each bin and for each species. As a result, a specific pattern of weights for the 1,300 bins was obtained and combined for all species in a weighted bin matrix.

For optimization, the spectral data were partitioned into 5 complementary subsets. One round of cross-validation involved a learning phase on 4 subsets (“training set”) and a validation of the predictive model on the remaining subset (“testing set”). Five rounds of cross-validation were performed by permutation, and the results from the five rounds combined.

To assess the accuracy of the database and calculate its performance in cross-validation, individual spectra were re-used as template for identification. The ASC algorithm compares the acquired spectrum to the specific pattern of each organism/organism group in the database and calculates a percent probability, or confidence value (%ID), which represents the similarity in terms of presence/absence of specific peaks between spectra. A perfect match provides a %ID of 99.9%. %ID >60 to 99.8% are considered as good. Scores <60% are considered to have no valid identification. The VITEK MS system renders the following types of identification results: “Single Choice”, when the spectrum acquired presents a high level of similarity (%ID >60 to 99.9%) with only one specific pattern in the database; “Low discrimination”, when the spectrum acquired presents a high level of similarity with 2 to 4 specific patterns in the database; or “No Identification”, when the spectrum acquired either does not match with any pattern in the database, or presents a high level of similarity to more than 4 specific patterns. During cross-validation, identification was considered as correct when the result was consistent with the reference identification. Low discrimination results were considered as correct if the expected identification was included in the matches. A misidentification was defined as discordant organism identification between the cross-validation result and the reference identification.

### Evaluation of performances by external validation

External spectra were generated from bacteria cultivated with different growth conditions (media, incubation time, etc) to mimic possible inter-laboratory variations. To reflect clinical laboratory practice, inactivated samples were spotted in duplicate, and analyzed with the updated database. If only one of the two spectra allowed a correct identification, the isolate was considered correctly identified. The cut-off for identification confidence was as described above.

## Results

To update the MALDI-TOF MS VITEK database, we used 84 *Brucella* strains, either reference strains or well characterized clinical/veterinary isolates (Tables 1 and S1), to generate independent spectra covering the *Brucella* genus. After initial selection based on quality criteria such as peak resolution, signal to noise ratio, number of peaks, absolute signal intensity, and intra-specific similarity, 590 spectra were retained and submitted for biomathematical analyses using an iterative system (bioMérieux patented ASC algorithm).

**Fig 1:**
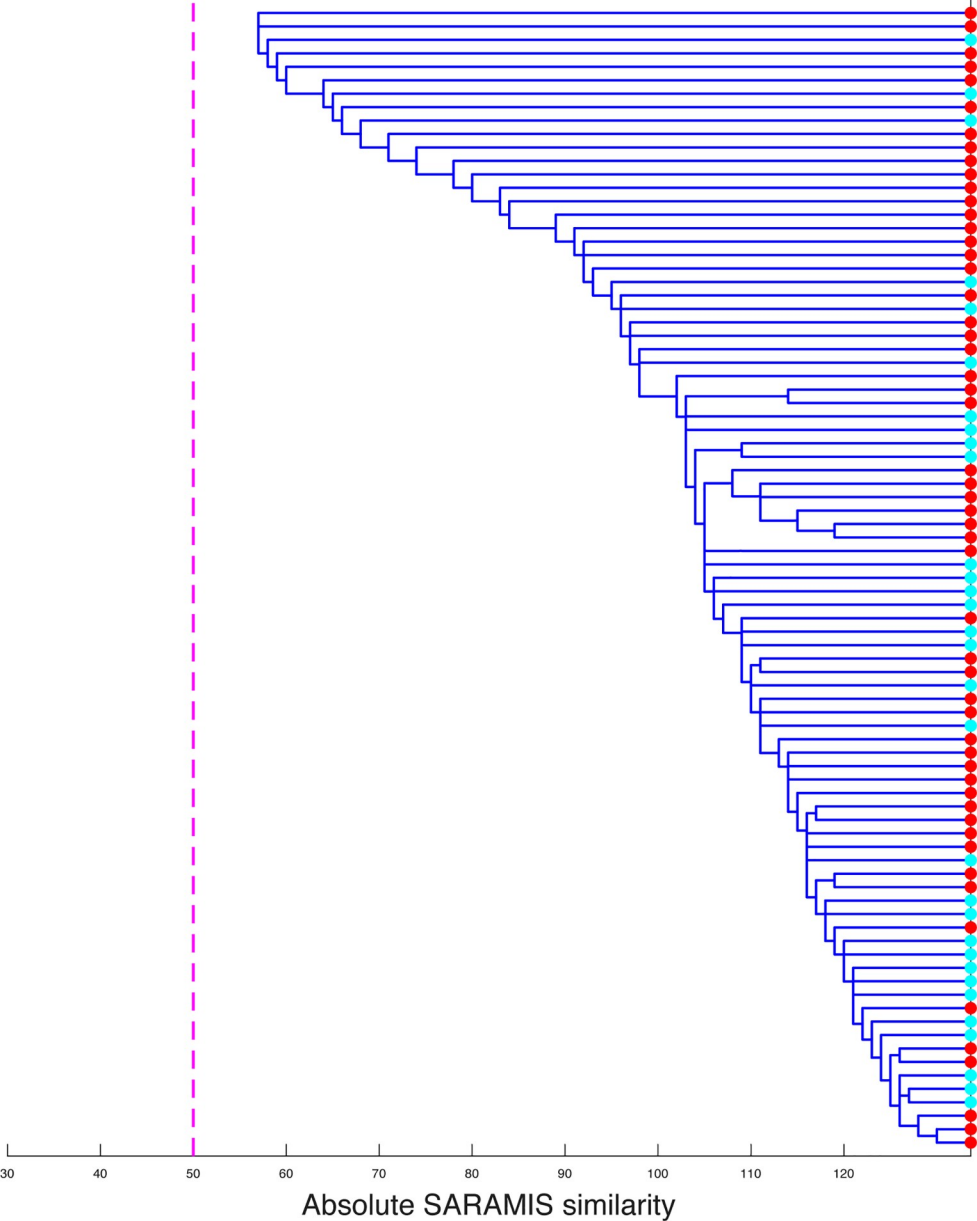
Proximity of *B*. *ceti* and *B*. *pinnipedialis* MALDI-TOF MS spectra. Cluster analysis, using correlation-based dissimilarity, was performed to assess the discriminating power of MALDI-TOF MS between the spectra corresponding to the species *B*. *ceti* (in red) or *B*. *pinnipedialis* (in blue). The threshold of 50 common peaks, which is considered as a minimum for considering that spectra are different, is shown as a dotted line.

Using an optimization process, we next evaluated the possibility to discriminate between different *Brucella* species and biovars. Discrimination between the different species was obtained, with the exception of *B*. *ceti* and *B*. *pinnipedialis*. These two species could not be clearly separated, as illustrated by the intertwining of their spectra on a dendrogram (Fig 1). Distinguishing the different biovars of *B*. *melitensis* and *B*. *abortus* was not possible. Discrimination between several of *B*. *suis* biovars was obtained (S1 Fig), but biovars 1 and 4 gave cross-identifications.

Classes representing the different *Brucella* species were thus created by grouping together the different biovars of *B*. *melitensis*, of *B*. *abortus* and of *B*. *suis*, and the two species *B*. *ceti* and *B*. *pinnipedialis*. The eight species represented in the MALDI-TOF database are thus: *B*. *melitensis* (biovar 1, 2 or 3), *B*. *abortus* (biovar 1, 2, 3, 4, 5, 6 or 9), *B*. *suis* (biovar 1, 2, 3, 4 or 5), *B*. *canis*, *B*. *ovis*, *B*. *ceti/B*. *pinnipedialis*, *B*. *inopinata* and *B*. *papionis*.

After optimization, cross validation was performed to evaluate the performance of the updated database, which contains 37,902 spectra covering 1,095 bacterial species including *Brucella*. This mathematical method is used to assess how accurately the database can perform. Correct identification at the genus level was obtained in 97.29% of cases (Table 3). Importantly, the remaining 2.71% of results corresponded to “no ID”, but never to an incorrect identification. At the species level, the performance varied between the different classes. For the three main zoonotic species (*B*. *melitensis*, *B*. *abortus* and *B*. *suis)*, correct identification was obtained with 96.06%, 100% or 89.34% of spectra, respectively.

**Table 3:** Results of identification in cross validation studies. Identifications (ID) results, either at genus or species/class level, were classified as correct (either “Single Choice” or “Low Discrimination”), discordant or no-identification (No ID). This table gives the % of each type of identification results for the indicated species/classes using the updated database.

CLASS	Species	# of strains	# of spectra	CORRECT ID		DISCORDANT ID		No ID	OVERALL % by genus	OVERALL % by class
				% Single choice	% Low discrimination	% Correct genus	% Incorrect genus			
***B*. *melitensis***	*B*. *melitensis*	5	22	90.91	4.55	0	0	4.55	**97.29**	**96.06**
	*B*. *melitensis* biovar 1	8	35	85.71	8.57	2.86	0	2.86		
	*B*. *melitensis* biovar 2	3	24	95.83	4.17	0	0	0		
	*B*. *melitensis* biovar 3	7	46	84.78	10.87	2.17	0	2.17		
***B*. *abortus***	*B*. *abortus* biovar 1	7	32	93.75	6.25	0	0	0		**100**
	*B*. *abortus* biovar 2	3	25	100	0	0	0	0		
	*B*. *abortus* biovar 3	7	36	100	0	0	0	0		
	*B*. *abortus* biovar 4	3	10	100	0	0	0	0		
	*B*. *abortus* biovar 5	1	7	100	0	0	0	0		
	*B*. *abortus* biovar 6	2	22	100	0	0	0	0		
	*B*. *abortus* biovar 9	2	24	100	0	0	0	0		
***B*. *suis***	*B*. *suis* biovar 1	7	47	89.36	2.13	0	0	8.51		**89.34**
	*B*. *suis* biovar 2	9	47	82.98	0	0	0	17.02		
	*B*. *suis biovar 3*	1	8	100	0	0	0	0		
	*B*. *suis* biovar 4	1	12	91.67	0	0	0	8.33		
	*B*. *suis* biovar 5	1	8	100	0	0	0	0		
***B*. *canis***	*B*. *canis*	5	19	84.21	15.79	0	0	0		**100**
***B*. *ovis***	*B*. *ovis*	4	30	93.33	6.67	0	0	0		**100**
***B*. *ceti/B*. *pinnipedialis***	*B*. *ceti*	8	54	98.15	1.85	0	0	0		**100**
	*B*. *pinnipedialis*	4	31	100	0	0	0	0		
***B*. *inopinata***	*B*. *inopinata*	2	23	95.65	4.35	0	0	0		**100**
***B*. *papionis***	*B*. *papionis*	2	28	100	0	0	0	0		**100**

Finally, as an external validation, the database was challenged with the MALDI-TOF spectra from 48 independent *Brucella* isolates, and 2 strains of *Ochrobactrum*, which are “near neighbors” of the *Brucella* genus (Tables 2 and S2).

The implemented database allowed correct identification at the genus level in 88.4% of cases, all the other results being “No-identification” but never misidentification as another genus (Tables 4 and 5). At the species level, the performances varied. For *B*. *melitensis*, *B*. *abortus*, and *B*. *suis*, correct identification was obtained for 100%, 92.3% or 100% of strains, respectively. It should be noted however that only one extra *B*. *suis* isolate was available to be tested in the external validation.

**Table 4:** Identification results in external validation. Identification (ID) results, either at genus or species/class level, were classified as correct, discordant or no-identification (No ID). This table gives the % of strains for which each type of identification result was obtained using the updated database. The number (n) of strains tested for each species/class is also indicated in parenthesis. N/A = not applicable.

		IDENTIFICATION AT THE GENUS LEVEL			IDENTIFICATION AT THE SPECIES LEVEL		
		% Correct ID	% Discordant ID	% No ID	% Correct ID	% Discordant ID	% No ID
*Brucella* species present in the database	*B*. *melitensis* (n = 16)	100% (16)	0% (0)	0% (0)	100% (16)	0% (0)	0% (0)
	*B*. *abortus* (n = 13)	92.3% (12)	0% (0)	7.7% (1)	92.3% (12)	0% (0)	7.7% (1)
	*B*. *suis* (n = 1)	100% (1)	0% (0)	0% (0)	100% (1)	0% (0)	0% (0)
	*B*. *canis* (n = 1)	100% (1)	0% (0)	0% (0)	0% (0)	100% (1)	0% (0)
	*B*. *ovis* (n = 3)	66.7% (2)	0% (0)	33.3% (1)	66.7% (2)	0% (0)	33.3% (1)
	*B*. *ceti/B*. *pinnipedialis* (n = 9)	66.7% (6)	0% (0)	33.3% (3)	66.7% (6)	0% (0)	33.3% (3)
	**Total (n = 43)**	**88,4% (38)**	**0% (0)**	**11.6% (5)**	**86% (37)**	**2.3% (1)**	**11.6% (5)**
*Brucella* species not present in the database	*B*. *microti* (n = 1)	0% (0)	0% (0)	100% (1)	N/A	N/A	N/A
	*B*. *neotomae* (n = 1)	0% (0)	0% (0)	100% (1)	N/A	N/A	N/A
*Brucella* strains with genus level characterization	*Brucella* spp (n = 3)	66.7% (2)	0% (0)	33.3% (1)	N/A	N/A	N/A
*Ochrobactrum* strains	*O*. *anthropi* (n = 1)	100% (1)	0% (0)	0% (0)	100% (1)	0% (0)	0% (0)
	*O*. *intermedium* (n = 1)	100% (1)	0% (0)	0% (0)	100% (1)	0% (0)	0% (0)

**Table 5:** Detailed results of external validation. For each strain, the identification results for the two deposits are given. %ID are confidence values given by the VITEK MS system for each identification result. COS-B = Columbia Blood Agar (bioMérieux, 43 041); BBA = *Brucella* blood agar (bioMérieux); COS-O = Columbia agar with 5% sheep blood (Oxoïd).

SAMPLE INFORMATION			IDENTIFICATION RESULTS			
Strain name	Species (biovar)	Culture condition	# of peaks	Identification type	Identification	% ID
75–3	*B*. *melitensis* (1)	COS-B (48h)	120	Single choice	*Brucella melitensis*	98.15
			99	Single choice	*Brucella melitensis*	99.99
78–158	*B*. *melitensis* (1)	COS-B (48h)	125	Single choice	*Brucella melitensis*	83.06
			118	Single choice	*Brucella melitensis*	99.95
88–44	*B*. *melitensis* (1)	COS-B (48h)	165	Single choice	*Brucella melitensis*	99.99
			141	Single choice	*Brucella melitensis*	99.99
1109	*B*. *melitensis* (1)	COS-B (48h)	121	Single choice	*Brucella melitensis*	99.99
			127	Single choice	*Brucella melitensis*	99.99
Rev1	*B*. *melitensis* (1)	COS-B (48h)	134	Single choice	*Brucella melitensis*	99.8
			136	Single choice	*Brucella melitensis*	99.99
16M+GFP	*B*. *melitensis* (1)	COS-B (48h)	129	Single choice	*Brucella melitensis*	99.99
			158	Single choice	*Brucella melitensis*	98.44
72–59	*B*. *melitensis* (3)	COS-B (48h)	150	Single choice	*Brucella melitensis*	98.1
			129	Single choice	*Brucella melitensis*	99.76
77–47	*B*. *melitensis* (3)	COS-B (48h)	127	Single choice	*Brucella melitensis*	99.99
			124	Single choice	*Brucella melitensis*	99.99
78–13	*B*. *melitensis* (3)	COS-B (48h)	111	Single choice	*Brucella melitensis*	63.65
			102	Single choice	*Brucella melitensis*	99.64
81–44	*B*. *melitensis* (3)	COS-B (48h)	122	No identification	* *	
			101	Single choice	*Brucella melitensis*	99.51
81–140	*B*. *melitensis* (3)	COS-B (48h)	148	Single choice	*Brucella melitensis*	99.99
			147	Single choice	*Brucella melitensis*	99.96
82–73	*B*. *melitensis* (3)	COS-B (48h)	99	Single choice	*Brucella melitensis*	87.07
			101	Single choice	*Brucella melitensis*	99.97
82–87	*B*. *melitensis* (3)	COS-B (48h)	121	Single choice	*Brucella melitensis*	99.79
			123	Single choice	*Brucella melitensis*	89.74
90–129	*B*. *melitensis* (3)	COS-B (48h)	110	Single choice	*Brucella melitensis*	99.99
			125	Single choice	*Brucella melitensis*	99.99
91–244	*B*. *melitensis* (3)	COS-B (48h)	91	No identification	* *	
			101	Single choice	*Brucella melitensis*	99.99
79–185	*B*. *melitensis* (3)	COS-B (48h)	163	Single choice	*Brucella melitensis*	89.68
			162	No identification	* *	
2308	*B*. *abortus* (1)	COS-B (48h)	165	Single choice	*Brucella abortus*	99.99
			154	Single choice	*Brucella abortus*	99.99
S19	*B*. *abortus* (1)	BBA (72h)	66	Low Discrimination	*Brucella abortus*	50.14
			80	Single choice	*Brucella abortus*	99.99
RB51	*B*. *abortus* (1)	BBA (96h)	173	No identification	* *	
			104	No identification	* *	
97-4775-11	*B*. *abortus* (1)	COS-B (48h)	134	Single choice	*Brucella abortus*	99.97
			136	Single choice	*Brucella abortus*	81.79
75–17	*B*. *abortus* (3)	COS-B (48h)	127	Single choice	*Brucella abortus*	99.99
			125	Single choice	*Brucella abortus*	99.99
83–227	*B*. *abortus* (3)	COS-B (48h)	137	Single choice	*Brucella abortus*	98.34
			101	Single choice	*Brucella abortus*	99.87
83–233	*B*. *abortus* (3)	COS-B (48h)	136	Single choice	*Brucella abortus*	99.99
			117	Single choice	*Brucella abortus*	99.99
79–153	*B*. *abortus* (3)	COS-B (48h)	130	Single choice	*Brucella abortus*	99.99
			136	Single choice	*Brucella abortus*	99.99
82–41	*B*. *abortus (3)*	COS-B (48h)	130	Single choice	*Brucella abortus*	99.99
			138	Single choice	*Brucella abortus*	99.99
99-9971-135	*B*. *abortus (7)*	COS-B (72h)	81	Single choice	*Brucella abortus*	99.99
			105	Single choice	*Brucella abortus*	99.99
03-4923-239-D	*B*. *abortus (7)*	COS-B (48h)	148	Single choice	*Brucella abortus*	99.99
			149	Single choice	*Brucella abortus*	99.99
77–9	*B*. *abortus (9)*	COS-B (48h)	94	Single choice	*Brucella abortus*	99.99
			118	Single choice	*Brucella abortus*	99.99
80–133	*B*. *abortus (9)*	COS-B (48h)	98	Single choice	*Brucella abortus*	99.99
			115	Single choice	*Brucella abortus*	87.95
04-1361Sisak-4	*B*. *suis (1)*	COS-O (72h)	87	Single choice	*Brucella suis*	99.99
			78	Single choice	*Brucella suis*	99.99
Mex 51	*B*. *canis*	COS-B (48h)	140	Single choice	*Brucella suis*	99.98
			158	No identification	* *	
76250	*B*. *ovis*	COS-B + 5% CO_2_ (96h)	125	No identification	* *	
			107	No identification	* *	
91268	*B*. *ovis*	COS-B + 5% CO_2_ (96h)	116	No identification	* *	
			104	Single choice	*Brucella ovis*	99.99
91212	*B*. *ovis*	COS-B + 5% CO_2_ (96h)	130	Single choice	*Brucella ovis*	99.99
			101	Single choice	*Brucella ovis*	99.96
F5/99	*B*. *ceti*	COS-B + 5% CO_2_ (96h)	106	No identification	* *	
			91	No identification	* *	
UK3/05	*B*. *ceti*	COS-B + 5% CO_2_ (96h)	127	No identification	* *	
			121	Single choice	*Brucella ceti/pinnipedialis*	99.98
UK9/99	*B*. *pinnipedialis*	COS-B + 5% CO_2_ (96h)	118	Single choice	*Brucella ceti/pinnipedialis*	83.28
			126	Single choice	*Brucella ceti/pinnipedialis*	99.99
36/94	*B*. *ceti*	COS-B + 5% CO_2_ (96h)	112	No identification	* *	
			119	No identification	* *	
49/94	*Brucella sp*. *(B*. *ceti *?*)*	COS-B + 5% CO_2_ (96h)	119	Single choice	*Brucella ceti/pinnipedialis*	99.99
			128	Single choice	*Brucella ceti/pinnipedialis*	99.96
02/611	*Brucella sp*. *(B*. *ceti-like)*	COS-B + 5% CO_2_ (96h)	148	Single choice	*Brucella ceti/pinnipedialis*	99.99
			133	Single choice	*Brucella ceti/pinnipedialis*	100
15/95	*Brucella sp*. *(B*. *pinnipedialis *?*)*	COS-B + 5% CO_2_ (96h)	112	No identification	* *	
			114	No identification	* *	
56/94	*B*. *pinnipedialis*	COS-B + 5% CO_2_ (96h)	116	No identification	* *	
			126	Single choice	*Brucella ceti/pinnipedialis*	99.77
96/408	*B*. *pinnipedialis*	COS-B + 5% CO_2_ (96h)	131	Single choice	*Brucella ceti/pinnipedialis*	99.99
			97	No identification	* *	
CCM4915	*B*. *microti*	COS-B (24h)	162	No identification	* *	
			181	No identification	* *	
5K33	*B*. *neotomae*	COS-B (48h)	145	No identification	* *	
			127	No identification	* *	
NF2637	*Brucella sp*.	COS-B (24h)	114	Single choice	*Brucella inopinata*	99.99
			144	Single choice	*Brucella inopinata*	77.86
NF2653	*Brucella sp*.	COS-B (24h)	141	No identification	* *	
			157	No identification	* *	
B13-0095	*Brucella sp*.	COS-B (24h)	136	Single choice	*Brucella inopinata*	100
			129	Single choice	*Brucella inopinata*	99.99
ATCC48188	*Ochrobactrum anthropi*	COS-B (48h)	127	Single choice	*Ochrobactrum anthropi*	100
			113	No identification	* *	
LMG3301	*Ochrobactrum intermedium*	COS-B (48h)	122	Single choice	*Ochrobactrum intermedium*	100
			118	Single choice	*Ochrobactrum intermedium*	100

Interestingly, the rare clinical isolate 02/611, described as *B*. *ceti*-like after molecular characterization [7], was indeed identified within the *B*.*ceti/B*. *pinnipedialis* class. Also, both the Bullfrog (B13-0095) and the Australian rodent (NF2637) isolates were identified as *B*. *inopinata*, in agreement with previous work showing that these belong to the atypical *Brucella* clade of this genus [27,28]. The two isolates belonging to “*B*. *abortus* biovar 7”, a rare biovar of this species, were identified as *B*. *abortus*. Finally, the recombinant 16M strain overexpressing the green fluorescent protein (GFP) was correctly identified as *B*. *melitensis* using this database. Moreover, using different culture conditions for 16M did not affect its identification by MALDI-TOF MS (S3 Table).

## Discussion

A major asset of this MALDI-TOF MS database is its ability to identify *Brucella* isolates at the species level, which is essential for following epidemiological outbreaks. Obtaining such a resolution was very challenging for this genus, as highlighted in previous studies [29], because of the high similarity between species at the genetic level [30]. Discrimination between species was made possible using a patented approach to differentiate closely related species using internal calibration and a two-step algorithm. This was not sufficient to distinguish the two species of *Brucella* from marine mammals (*B*. *ceti* and *B*. *pinnipedialis)*. This is in agreement with a recent Multi-Locus Sequence Analysis (MLSA) showing that the taxonomy is inconsistent with the phylogeny of these two species, and that taxonomic rearrangement should be envisaged [31]. This MALDI-TOF MS database is however able to discriminate eight different *Brucella* species, which include the most common in human or animal disease.

The updated database allowed correct identification of *Brucella* isolates at the genus level in 88.4% of cases. It is important to mention that none of them was identified as *Ochrobactrum spp*., a misidentification that is common with other standard identification methods [32–34] and recently reported using the VITEK MS database currently available [35]. Analysis at the species level gave only one discordant result, corresponding to cross identification between two *Brucella* species. Such result would have no consequence for human medicine, as identification at the genus level is sufficient to prescribe the appropriate treatment. As for all MALDI-TOF databases, the limitation of this system is its inability to identify non-clinically validated species or species not included in the database. However, the large coverage of the *Brucella* genus (in particular the most common species) in this database makes this risk is very minor.

Diminution of the performance at the genus and/or species level was due to “no ID” results for some rare and/or atypical *Brucella* spp. (*B*. *neotomae* strain 5K33, *B*. *microti* strain CCM4915, and the rodent isolate NF2653), several strains from marine mammals, and the vaccine strain *B*. *abortus* RB51. These results were not due to the quality of MALDl-TOF spectra, which was good (based on the number of spectral peaks, Table 5). In the spectra for RB51, we found that several masses characteristics of the *B*. *abortus* class were less frequently present, in particular the masses of 5,920.63, 6,040.32 and 7,467.89 Da were present in only 14.3% of spectra (*vs*. in 75–95% of the spectra of other *B*. *abortus* isolates, with a tolerance of 800 ppm). The only discordant result in our assay was obtained with *B*. *canis* Mex51, which was identified as *B*. *suis*. This was due to the presence in its spectra of additional masses that are common with the *B*. *suis* class in addition to the major peaks characteristics of the *B*. *canis* class. This finding is consistent with an exhaustive MLSA showing that *B*. *canis* strains are very close to *B*. *suis* biovars 3 and 4 [31].

Importantly, the MALDI-TOF database allowed the correct identification as *Brucella* of several recently discovered “atypical” isolates [5,6,28,36]. These strains represent a serious problem for diagnosis laboratories, as they are not identified as *Brucella* using classical phenotypic tests. It is possible that similar strains have been isolated in the past but misidentified. Very little is known concerning the ability of these new species to cause disease in humans or livestock. The possibility to identify these isolates as *Brucella* will thus be important for both human and animal health.

Overexpression of an exogenous protein (GFP) did not affect the identification of *B*. *melitensis* 16M. This is important since recombinant *Brucella* strains are common tools in research laboratories and could potentially infect lab workers. Moreover, the use of such *Brucella* strains as vaccines was proposed, since the presence anti-GFP antibodies would allow distinguishing vaccinated animals from naturally infected ones [37].

In conclusion, this updated MALDI-TOF MS database is a new diagnostic tool that allows the identification of *Brucella*. It combines precision of identification (broad coverage of the *Brucella* genus together with species-level identification) and widespread availability. After integration in the VITEK MS (v3.2), this will be the first *Brucella* database validated for diagnostic with CE accreditation and accessible to all users in routine. This will allow accurate diagnosis and timely treatment in brucellosis. These highly infectious pathogens also causing one of the most frequent laboratory-acquired infection [38], their rapid identification by MALDI-TOF MS will decrease the risk of accidental infection of laboratory workers. A paradox of global health however is that the countries where brucellosis is endemic may not have access to MALDI-TOF MS. This could be circumvented by the use of the in-tube inactivation method described earlier [19], which will allow the shipment of erstwhile infectious samples to mass spectrometry platforms.

## Supporting information

S1 Fig Discrimination between the different *B*. *suis* biovars. Multidimensional Scaling (MDS) analysis of MALDI-TOF spectra obtained with *B*. *suis* isolates. The similarity between spectra is represented as distances, which depend on the presence/absence of peaks and their intensity in compared spectra. Results are presented on the three first dimensions. The color code used for each biovar is indicated in the figure.
(TIF)

S1 Table Detailed list of the *Brucella* strains used to construct the MALDI-TOF MS database. Strains highlighted in grey are reference or type strains. The different culture conditions used for each strain (time of incubation in hours, media, ± 5% CO2) are indicated. BAS = *Brucella* blood agar with 5% sheep blood, Hemin and Vitamin K1 (Becton Dickinson PA-255509.05A), BBA = *Brucella* blood agar (bioMérieux, 411 968), CHOC-H = Chocolate agar (Hardy Diagnostic, E14), COS-B = Columbia Blood Agar (bioMérieux, 43 041), COS-D = Columbia agar with 5% sheep blood (Becton Dickinson, 90006 166), COS-O = Columbia agar with 5% sheep blood (Oxoïd, PB5039A).
(DOCX)

S2 Table Detailed list of bacterial strains and isolates used for the external validation of the database. (DOCX)

S3 Table Identification results on *B*. *melitensis* strain 16M cultivated with different conditions. The culture conditions (media, time of incubation in hours, ± 5% CO_2_) are indicated. BAS = *Brucella* agar with 5% sheep blood, hemin & vitamin K1 (Becton Dickinson); BBA = *Brucella* blood agar (bioMérieux); CHOC-O = Chocolate agar plate with vitox (Oxoïd); COS-B = Columbia Blood Agar (bioMérieux, 43 041); COS-O = Columbia agar with 5% sheep blood (Oxoïd); MHB = Mueller Hinton agar with 5% sheep blood (Biorad); MHF = Mueller Hinton agar with 5% horse blood and β-NAD (Biorad); TSA-S = Trypticase soy agar with 5% sheep blood (Becton Dickinson).
(DOCX)
