# Supplementary figures and images for: A MALDI-TOF MS database with broad genus coverage for species-level identification of Brucella
Source: PLoS Negl Trop Dis. 2018 Oct 18;12(10):e0006874. doi: 10.1371/journal.pntd.0006874 (PMC6207331; doi:10.1371/journal.pntd.0006874)

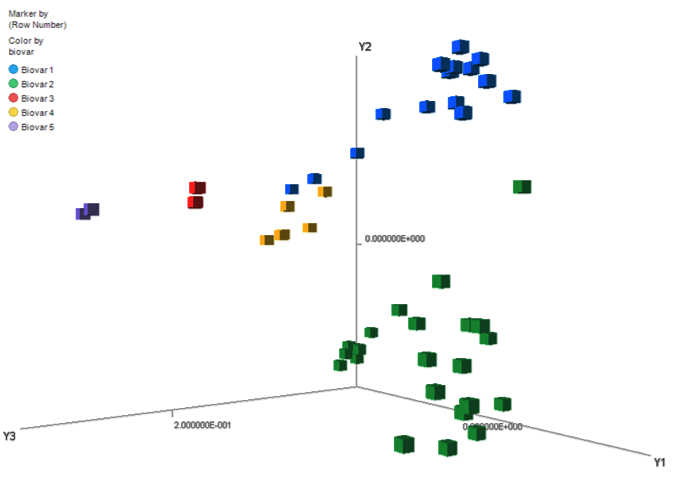

Supplement: S1 Fig — Multidimensional Scaling (MDS) analysis of MALDI-TOF spectra obtained with B. suis isolates. The similarity between spectra is represented as distances, which depend on the presence/absence of peaks and their intensity in compared spectra. Results are presented on the three first dimensions. The color code used for each biovar is indicated in the figure. (TIF) [file pntd.0006874.s001.tif]
