# Supplementary material for: A MALDI-TOF MS database with broad genus coverage for species-level identification of Brucella
Source: PLoS Negl Trop Dis. 2018 Oct 18;12(10):e0006874. doi: 10.1371/journal.pntd.0006874 (PMC6207331; doi:10.1371/journal.pntd.0006874)
Supplement: S1 Table — Strains highlighted in grey are reference or type strains. The different culture conditions used for each strain (time of incubation in hours, media, ± 5% CO2) are indicated. BAS = Brucella blood agar with 5% sheep blood, Hemin and Vitamin K1 (Becton Dickinson PA-255509.05A), BBA = Brucella blood agar (bioMérieux, 411 968), CHOC-H = Chocolate agar (Hardy Diagnostic, E14), COS-B = Columbia Blood Agar (bioMérieux, 43 041), COS-D = Columbia agar with 5% sheep blood (Becton Dickinson, 90006 166), COS-O = Columbia agar with 5% sheep blood (Oxoïd, PB5039A). (DOCX) [file pntd.0006874.s002.docx]

**Table S1**

| **Strain/isolate name** | ***Brucella* species** | **Biovar** | **Description** | **Culture conditions** |
| --- | --- | --- | --- | --- |
| 16M | *B. melitensis* | 1 | *Brucella melitensis* biovar 1 reference strain (ATCC 23456). Isolated from a goat in USA. | 48h on BAS, BBA, CHOC-H, COS-B, COS-O or COS-D |
| 95-5009-1 | *B. melitensis* | 1 | Veterinary isolate. Isolated in France (Dept. 67) in 1995 from a sheep. | 72h on BBA, COS-B or COS-O |
| 95-2426-961 | *B. melitensis* | 1 | Veterinary isolate. Isolated in France (Dept. 67) in 1995 from a sheep (retromammary lymph nodes) in a herd with multiple abortions (20% serologic prevalence). | 72h on BBA, COS-B or COS-O |
| 13-2582-4590 | *B. melitensis* | 1 | Clinical isolate (Indian type). Isolated in France in 2013 from a patient from Kazakztan. | 72h on BBA, COS-B or COS-O |
| 05-0737 | *B. melitensis* | 1 | Clinical isolate (fuschin sensitive). Isolated in France in 2005 from a 59-yr old patient (blood culture) from Turkey. | 72h on BBA, COS-B or COS-O |
| 08-2437-5214 | *B. melitensis* | 1 | Veterinary isolate (field isolate of the Rev1 vaccine strain). Isolated in France (Dept. 04) in 2008 from a caprine. | 96h on BBA or COS-B |
| 63/9 | *B. melitensis* | 2 | *Brucella melitensis* biovar 2 reference strain (ATCC 23457). Isolated from a patient (blood and bone marrow). | 48h on BAS, BBA, COS-B or COS-O |
| 11-939-2005 | *B. melitensis* | 2 | Clinical isolate. Isolated in France in 2011 from a 53-yr old male patient (blood culture) originating from Kuwait. | 96h on BBA or COS-B |
| 04-1553 | *B. melitensis* | 2 | Clinical isolate. Isolated in France in 2004 from a 11-yr old female patient (blood culture) who travelled in Lebanon in 2003. | 72h on BBA, COS-B or COS-O |
| Ether | *B. melitensis* | 3 | *Brucella melitensis* biovar 3 reference strain (ATCC 23458). Clinical isolate. | 48h on BAS, BBA, COS-B or COS-O |
| 11-441-982 | *B. melitensis* | 3 | Clinical isolate. Isolated in France in 2011 from a 56-yr old patient (blood culture) with history of raw sheep cheese consumption in Portugal. | 96h on BBA or COS-B |
| 10-394 | *B. melitensis* | 3 | Clinical isolate. Isolated in France in 2010 from a 58-yr old male patient (blood culture) who is farmer in Algeria. | 96h on BBA or COS-B |
| 07-1184-2852 | *B. melitensis* | 3 | Clinical isolate. Isolated in France in 2007 from a 45-yr old male patient (blood culture) with recent history of raw cheese consumption in Sicily (Italy). | 72h on BBA, COS-B or COS-O |
| 02-1213 | *B. melitensis* | 3 | Veterinary isolate. Isolated in France (Dept. 64) in 2002 from a bovine placenta after spontaneous abortion (7 months). | 72h on BBA, COS-B or COS-O |
| 05-0682 | *B. melitensis* | 3 | Clinical isolate. Isolated in France in 2005 from a 17-yr old male patient (blood culture) travelling back from Turkey. | 72h on BBA, COS-B or COS-O |
| 11-2159-4003 | *B. melitensis* | 3 | Clinical isolate (fuschin sensitive). Isolated in France in 2011 from a 62-yr old female patient (blood culture) with recent history of raw cheese consumption in Portugal. | 96h on BBA or COS-B |
| B115 | *B. melitensis* |  | Veterinary isolate (naturally attenuated rough strain). Isolated from a goat in Malta. | 72h on BBA, COS-B or COS-O |
| BT020216 | *B. melitensis* |  | Clinical isolate. Isolated in USA. | 48h on BAS, CHOC-H, COS-B or COS-D |
| BT071315-0001 | *B. melitensis* |  | Clinical isolate. Isolated in USA. | 48h on BAS, CHOC-H, COS-B or COS-D |
| BT072914 | *B. melitensis* |  | Clinical isolate. Isolated in USA. | 48h on BAS, CHOC-H, COS-B or COS-D |
| BT1202150001 | *B. melitensis* |  | Clinical isolate. Isolated in USA. | 48h on BAS, CHOC, COS-B or COS-D |
| 544 | *B. abortus* | 1 | *Brucella abortus* biovar 1 reference strain (ATCC 23448). Isolated from a bovine (*Bos taurus*) in UK. | 48h on BAS, BBA, CHOC-H, COS-B, COS-O or COS-D |
| 01-673 | *B. abortus* | 1 | Veterinary isolate. Isolated in France (Dept. 64) in 2001 from a bovine (iliac ganglion). | 96h on BBA or COS-B |
| 03-2770-3 | *B. abortus* | 1 | Veterinary isolate (strain RG 513/02a). Isolated in Brazil in 2003 from a bovine. | 96h on BBA or COS-B |
| 05-147-200 | *B. abortus* | 1 | Veterinary isolate. Isolated in Japan in 2004 from a bovine (milk). | 72h on BBA, COS-B or COS-O |
| 93-12101 | *B. abortus* | 1 | Clinical isolate. Isolated in Constantine (Algeria) in 1993 from a 65-yr old male patient (blood culture) who is a farm worker. | 96h on BBA or COS-B |
| 2000031295 | *B. abortus* | 1 | Clinical isolate. Isolated in USA. | 48h on BAS, CHOC-H, COS-B or COS-D |
| 86/8/59 | *B. abortus* | 2 | *Brucella abortus* biovar 2 reference strain (ATCC 23449). Isolated from a bovine fetus. | 48h on BAS, BBA, CHOC-H, COS-B, COS-O or COS-D |
| 03-2770-11 | *B. abortus* | 2 | Veterinary isolate (strain RG 629/03a). Isolated in Brazil in 2003 from a bovine. | 96h on BBA or COS-B |
| 92-601 | *B. abortus* | 2 | Veterinary isolate. Isolated in France (Dept. 65) in 1992 from a bovine aborted fetus. | 96h on BBA or COS-B |
| Tulya | *B. abortus* | 3 | *Brucella* abortus biovar 3 reference strain (ATCC 23450). Clinical isolate. | 48h on BAS, BBA, COS-B or COS-O |
| 12-1745 | *B. abortus* | 3 | Veterinary isolate. Isolated in France (Dept. 59) in 2012 from a bovine. | 96h on BBA or COS-B |
| 03-2055 | *B. abortus* | 3 | Veterinary isolate. Isolated in France (Dept. 03) in 2003 from an ovine (vaginal swab). | 96h on BBA or COS-B |
| 99-4566 | *B. abortus* | 3 | Veterinary isolate. Isolated in France (Dept. 53) in 1999 from a bovine after spontaneous abortion at 7 months. | 96h on BBA or COS-B |
| 03-4278 | *B. abortus* | 3 | Clinical isolate (negative oxidase, characteristic of Africain strains of *B. abortus* bv 3). Isolated in France in 2003 from a 49-yr old male patient (blood culture) travelling in Mauritania for 3 months (in contact in animals in slaughterhouse). | 96h on BBA or COS-B |
| 92-7369-2 | *B. abortus* | 3 | Veterinary isolate (negative oxidase, characteristic of Africain strains of *B. abortus* bv 3). Isolated in Conakry (Guinea) in 1992 from a bovine hygroma. | 96h on BBA or COS-B |
| 292 | *B. abortus* | 4 | *Brucella abortus* biovar 4 reference strain (ATCC 23451). Isolated from a bovine fetus. | 48h on BAS, CHOC-H, COS-B or COS-D, or 96h on BBA or COS-B |
| 99-9473 | *B. abortus* | 4 | Veterinary isolate. Isolated in France (Dept. 43) in 1999 from a bovine. | 96h on BBA or COS-B |
| B3196 | *B. abortus* | 5 | *Brucella abortus* biovar 5 reference strain (ATCC 23452). Isolated from a bovine after uterine discharge. | 48h on BAS, CHOC-H, COS-B or COS-D or 72h on BBA, COS-B or COS-O |
| 870 | *B. abortus* | 6 | *Brucella abortus* biovar 6 reference strain (ATCC 23453). Isolated from a bovine fetus. | 48h on BAS, BBA, CHOC-H, COS-B, COS-O or COS-D |
| C68 | *B. abortus* | 9 | *Brucella abortus* biovar 9 reference strain (ATCC 23455). Isolated from a bovine fetus. | 48h on BAS, BBA, CHOC-H, COS-B, COS-O or COS-D |
| 1330 | *B. suis* | 1 | *Brucella suis* biovar 1 reference strain (ATCC 23444). Isolated from a swine in USA. | 48h on BAS, BBA, CHOC-H, COS-B, COS-O or COS-D |
| 13-896-1815 | *B. suis* | 1 | Clinical isolate. Isolated in Papeete (French polynesia) in 2013. | 72h on BBA, COS-B or COS-O |
| 12-2826-5972 | *B. suis* | 1 | Clinical isolate. Isolated in Noumea (New Caledonia) in 2012. | 72h on BBA, COS-B or COS-O |
| 04-1361Djakovo-1 | *B. suis* | 1 | Veterinary isolate. Isolated in Djakovo (Croatia) in 2004 from a wild boar. | 72h on BBA, COS-B or COS-O |
| 11-2920-5143 | *B. suis* | 1 | Clinical isolate (fuschin resistant, FII air and CO_2_). Isolated in France in 2011 from a 81-yr old female patient (blood culture) living in Buenos Aires 6 months/year. | 72h on BBA, COS-B or COS-O |
| 05-4266 | *B. suis* | 1 | Clinical isolate (fuschin resistant with CO_2_). Isolated in Wallis et Futuna in 2005 from a 28-yr old female patient (blood culture). | 72h on BBA, COS-B or COS-O |
| Thompsen | *B. suis* | 2 | *Brucella suis* biovar 2 reference strain (ATCC 23445). Isolated from a hare. | 48h on BAS, BBA, COS-B or COS-O |
| 12-4327-8815 | *B. suis* | 2 | Veterinary isolate. Isolated in France (Dept. 03) in 2010 from a hare. | 72h on BBA, COS-B or COS-O |
| 12-2885-6046 | *B. suis* | 2 | Clinical isolate. Isolated in France (Dept. 69) in 2012. | 96h on BBA or COS-B |
| 11-3301-6219 | *B. suis* | 2 | Veterinary isolate. Isolated in France (Dept. 18) in 2011 from a swine. | 96h on BBA or COS-B |
| 11-2942-5156 | *B. suis* | 2 | Veterinary isolate. Isolated in France (Dept. 73) in 2011 from a wild boar (testis). | 96h on BBA or COS-B |
| 11-028-111 | *B. suis* | 2 | Veterinary isolate. Isolated in France (Dept. 65) in 2010 from a swine (vaginal swab). | 96h on BBA or COS-B |
| 09-372-779 | *B. suis* | 2 | Veterinary isolate. Isolated in France (Dept. 26) in 2009 from an ovine. | 96h on BBA or COS-B |
| 05-3495 | *B. suis* | 2 | Clinical isolate. Isolated in France in 2005 from a 42-yr old male hunter (hip prosthesis infection). | 72h on BBA, COS-B or COS-O |
| 00-4898 | *B. suis* | 2 | Veterinary isolate. Isolated in France (Dept. 63) in 2000 from a bovine. | 96h on BBA or COS-B |
| 686 | *B. suis* | 3 | *Brucella suis* biovar 3 reference strain (ATCC 23446). Isolated from a Reindeer in Alaska. | 48h on BAS, BBA, COS-B or COS-O |
| 40 | *B. suis* | 4 | *Brucella suis* biovar 4 reference strain (ATCC 23447). Isolated from a Reindeer in Russia. | 48h on BAS, BBA, COS-B or COS-O |
| 513 | *B. suis* | 5 | *Brucella suis* biovar 5 reference strain (NCTC 11996). Isolated from a wild rodent in ex-USSR. | 48h on BAS, BBA, COS-B or COS-O |
| 03-2770-12 | *B. canis* |  | Veterinary isolate (fuschin +). Isolated in Brazil in 2003 from a dog. | 72h on BBA, COS-B or COS-O |
| 04-2330-1 | *B. canis* |  | Veterinary isolate (fuschin resistant). Isolated in Belgrade (Serbia) in 2004 from a dog (blood culture). | 72h on BBA, COS-B or COS-O |
| 09-369-776(2) | *B. canis* |  | Veterinary isolate. Isolated in Helsinki (Finland) in 2009 from a dog. | 72h on BBA or COS-B |
| 11-1961-3694(1) | *B. canis* |  | Veterinary isolate (fuschin resistant). Isolated in Sueden in 2011 from a 3-yr female dog (placenta) with abortion and history of travelling in Poland and Czech republic | 72h on BBA, COS-B or COS-O |
| 08-1276-2270 | *B. canis* |  | Clinical isolate (fuschin +). Isolated in Papeete (French Polynesia) in 2008 from a 3-yr old female patient (blood culture). | 72h on BBA, COS-B or COS-O |
| 63/290 | *B. ovis* |  | *Brucella ovis* type strain (ATCC 25840). Isolated from a sheep in Australia. | 48h on BAS, BBA, COS-B or COS-O (+ 5% CO_2_) |
| 12-1497-b | *B. ovis* |  | Veterinary isolate. Isolated in France (Dept. 04) in 2012 from an ovine. | 48h on BAS, BBA, COS-B or COS-O (+ 5% CO_2_) |
| 11-868-1991 | *B. ovis* |  | Veterinary isolate. Isolated in France (Dept. 13) in 2011 from an ovine. | 48h on BBA or COS-B or 96h on BAS (+ 5% CO_2_) |
| 12-3480-79 | *B. ovis* |  | Veterinary isolate. Isolated in France (PACA region) in 2012 from a ram. | 48h on BBA or COS-B or 96h on BAS (+ 5% CO_2_) |
| B1/94 | *B. ceti* |  | *Brucella ceti* reference strain (NCTC 12891). Isolated from a harbor porpoise in UK | 48h on BAS, BBA, COS-B or COS-O or 96h on BBA or COS-B (+ 5% CO_2_) |
| 34/94 | *B. ceti* |  | Veterinary isolate. Isolated in Scotland from a harbour porpoise (*Phocoena phocoena*). | 48h on BBA or COS-B or 96h on BAS (+ 5% CO_2_) |
| 97/0776 | *B. ceti* |  | Veterinary isolate (also known as 7763/2). Isolated on the Altantic coast of France (Dept. 44) in 1997 from a bottlenose dolphin (*Tursiops truncatus*). | 48h on BBA or COS-B or 96h on BAS (+ 5% CO_2_) |
| B202R | *B. ceti* |  | Veterinary isolate. Identified in North Altantic (Norway) from the liver of a common mink whale (*Balænoptera acutorostrata*) | 48h on BBA or COS-B or 96h on BAS (+ 5% CO_2_) |
| 47/94 | *B. ceti* |  | Veterinary isolate. Isolated in Scotland from a common dolphin (*Delphinus delphis*) | 48h on BBA or COS-B or 96h on BAS (+ 5% CO_2_) |
| B14/94 | *B. ceti* |  | Veterinary isolate (also known as M644/93/1). Isolated in Scotland from a common dolphin (*Delphinus delphis*). | 48h on BAS, BBA, COS-B or COS-O (+ 5% CO_2_) |
| 98/230 | *B. ceti* |  | Veterinary isolate (also known as NVSL-98-0230). Isolated in 1998 from a lung necropsy specimen (nodule within a granulomatous focus) of a 21 years old female bottlenose dolphin (*Tursiops truncatus*) that died acutely on Dec 1997 (US Navy, California, USA) [1]. | 48h on BBA or COS-B or 96h on BAS (+ 5% CO_2_) |
| 5/95 | *B. ceti* |  | Veterinary isolate. Isolated from a striped dolphin (*Stenella coetuleoalba*) | 48h on BBA or COS-B or 96h on BAS (+ 5% CO_2_) |
| B2/94 | *B. pinnipedialis* |  | *Brucella pinnipedialis* reference strain (NCTC 12890). Isolated from a common seal in Scotland | 48h on BAS, BBA, COS-B or COS-O or 96h on BAS or COS-B (+ 5% CO_2_) |
| 39/94 | *B. pinnipedialis* |  | Veterinary isolate (also known as M292/94/1). Isolated in Scotland from a common seal (*Phoca vitulina*). | 48h on BAS, BBA, COS-B or COS-O (+ 5% CO_2_) |
| 55/94 | *B. pinnipedialis* |  | Veterinary isolate. Isolated in UK from an otter (*Lutra lutra*) found dead as a road casualty. | 48h on BBA or COS-B or 96h on BAS (+ 5% CO_2_) |
| 61/94 | *B. pinnipedialis* |  | Veterinary isolate. Isolated in UK from a grey seal (*Halichoerus grypus*). Whatmore. BMC. 2007] | 48h on BBA or COS-B or 96h on BAS (+ 5% CO_2_) |
| BO1 | *B. inopinata* |  | *Brucella inopinata* type strain (BCCN 09-01). Clinical isolate. Isolated from a 71-yr old female patient (breast implant infection) in USA [2]. | 48h on BAS, BBA, COS-B or COS-O |
| BO2 | *B. inopinata*-like |  | Clinical isolate. Isolated in Australia from a lung biopsy in a 52-yr old patient with chronic destructive pneumonia [3]. | 48h on BAS, BBA, COS-B or COS-O |
| F8/08-60 | *B. papionis* |  | *Brucella papionis* type strain (NVSL 07-0026-1). Isolated in USA in 2006 from the postpartum uterus of a 13-yr old baboon (*Papio* spp) that had stillbirth and retained placenta (animal facility of SNPRC,Texas) [4]. | 48h on BAS, BBA, COS-B or COS-O or 96h on BAS or COS-B |
| F8/08-61 | *B. papionis* |  | Veterinary isolate (also named NVSL 07-0224-1). Isolated in USA in 2007 from the uterus of a 8-yr old baboon (*Papio* spp) that had stillbirth (animal facility of SNPRC,Texas) [4]. | 48h on BAS, BBA, COS-B or COS-O or 96h on BAS or COS-B |

References :

1. Miller WG, Adams LG, Ficht TA, Cheville NF, Payeur JP, Harley DR, et al. *Brucella*-induced abortions and infection in bottlenose dolphins (*Tursiops truncatus*). J Zoo Wildl Med. 1999;30: 100–10.

2. De BK, Stauffer L, Koylass MS, Sharp SE, Gee JE, Helsel LO, et al. Novel *Brucella* Strain (BO1) Associated with a Prosthetic Breast Implant Infection. J Clin Microbiol. 2008;46: 43–49. doi:10.1128/JCM.01494-07

3. Tiller R V, Gee JE, Lonsway DR, Gribble S, Bell SC, Jennison A V, et al. Identification of an unusual *Brucella* strain (BO2) from a lung biopsy in a 52 year-old patient with chronic destructive pneumonia. BMC Microbiol. 2010;10: 23. doi:10.1186/1471-2180-10-23

4. Schlabritz-Loutsevitch NE, Whatmore AM, Quance CR, Koylass MS, Cummins LB, Dick Jr EJ, et al. A novel *Brucella* isolate in association with two cases of stillbirth in non-human primates - first report. J Med Primatol. 2009;38: 70–73. doi:10.1111/j.1600-0684.2008.00314.x

5. Garin-Bastuji B, Mick V, Le Carrou G, Allix S, Perrett LL, Dawson CE, et al. Examination of taxonomic uncertainties surrounding *Brucella abortus* bv. 7 by phenotypic and molecular approaches. Appl Environ Microbiol. 2014;80: 1570–9. doi:10.1128/AEM.03755-13

6. Whatmore AM, Dawson C, Muchowski J, Perrett LL, Stubberfield E, Koylass M, et al. Characterisation of North American *Brucella* isolates from marine mammals. Roop RM, editor. PLoS One. 2017;12: 1–17. doi:10.1371/journal.pone.0184758

7. Ewalt DR, Payeur JB, Martin BM, Cummins DR, Miller WG. Characteristics of a *Brucella* Species from a Bottlenose Dolphin (*Tursiops Truncatus*). J Vet Diagnostic Investig. 1994;6: 448–452. doi:10.1177/104063879400600408

8. Groussaud P, Shankster SJ, Koylass MS, Whatmore AM. Molecular typing divides marine mammal strains of *Brucella* into at least three groups with distinct host preferences. J Med Microbiol. 2007;56: 1512–1518. doi:10.1099/jmm.0.47330-0

9. Scholz HC, Hubalek Z, Sedláček I, Vergnaud G, Tomaso H, Al Dahouk S, et al. *Brucella microti* sp. nov., isolated from the common vole *Microtus arvalis*. Int J Syst Evol Microbiol. 2008;58: 375–382. doi:10.1099/ijs.0.65356-0

10. Stoenner HG, Lackman DB. A new species of *Brucella* isolated from the desert wood rat, *Neotoma lepida Thomas*. Am J Vet Res. 1957;18: 947–51.

11. Tiller R V, Gee JE, Frace MA, Taylor TK, Setubal JC, Hoffmaster AR, et al. Characterization of novel *Brucella* strains originating from wild native rodent species in North Queensland, Australia. Appl Environ Microbiol. 2010;76: 5837–5845. doi:10.1128/AEM.00620-10

12. McDonald WL, Jamaludin R, Mackereth G, Hansen M, Humphrey S, Short P, et al. Characterization of a *Brucella* sp. strain as a marine-mammal type despite isolation from a patient with spinal osteomyelitis in New Zealand. J Clin Microbiol. 2006;44: 4363–4370. doi:10.1128/JCM.00680-06

13. Soler-Lloréns PF, Quance CR, Lawhon SD, Stuber TP, Edwards JF, Ficht TA, et al. A *Brucella* spp. Isolate from a Pac-Man Frog (*Ceratophrys ornata*) Reveals Characteristics Departing from Classical Brucellae. Front Cell Infect Microbiol. 2016;6: 116. doi:10.3389/fcimb.2016.00116
