# Supplementary material for: A MALDI-TOF MS database with broad genus coverage for species-level identification of Brucella
Source: PLoS Negl Trop Dis. 2018 Oct 18;12(10):e0006874. doi: 10.1371/journal.pntd.0006874 (PMC6207331; doi:10.1371/journal.pntd.0006874)
Supplement: S2 Table — (DOCX) [file pntd.0006874.s003.docx]

**Table S2**

| **Strain/isolate** | **Species** | **Biovar** | **Description** |
| --- | --- | --- | --- |
| 2308 | *B. abortus* | 1 | Highly virulent strain recovered in 1940 from an aborted fetus of a cow that had been in contact with cattle experimentally infected with a mixture of *B. abortus* cultures. Widely used within the brucellosis research community. |
| S19 | *B. abortus* | 1 | Vaccine strain S19 (spontaneously attenuated strain; used as a vaccine for the control of cattle brucellosis). |
| RB51 | *B. abortus* | 1 | Vaccine strain (genetically stable. rough morphology mutant). Derived from strain 2308. Rifampicin resistant. |
| 97-4775-11 | *B. abortus* | 1 | Isolated from a bovine in 1997 (France, Dept. 54) |
| 75-17 | *B. abortus* | 3 | Isolated from a cow (France, Dept.35) |
| 83-227 | *B. abortus* | 3 | Isolated from a bovine (Rwanda) |
| 83-233 | *B. abortus* | 3 | Isolated from a bovine (Niger) |
| 79-153 | *B. abortus* | 3 | Isolated from a bovine (Senegal) |
| 82-41 | *B. abortus* | 3 | Isolated from a bovine (Guinée-Bissau, Africa) |
| 99-9971-135 | *B. abortus* | 7 | Isolated in a bovine in 1988 (Mongolia). Proposed new reference strain for *B. abortus* biovar 7 [5] |
| 03-4923-239-D | *B. abortus* | 7 | Isolated in a bovine in 2003 (Turkey) [5] |
| 77-9 | *B. abortus* | 9 | Isolated from a cow (Region Nord, France) |
| 80-133 | *B. abortus* | 9 | Isolated from a dog (Belgium) |
| Mex 51 | *B. canis* |  | Isolated from a dog lymph node (Mexico). Also identified as NCTC11365 |
| 36/94 | *B. ceti* |  | Isolated from a harbour porpoise (Inverness, Scotland) [6] |
| F5/99 | *B. ceti* |  | Obtained from the aborted fetus of a bottlenose dolphin (*Tursiops truncatus*) in California in 1992. Also known as NVSL-92-1350 [7] |
| UK3/05 | *B. ceti* |  | Isolated from a striped dolphin (*Stenella coeruleoalba*) in Scotland. Also known as M13/05/1 [8] |
| 75-3 | *B. melitensis* | 1 | Clinical isolate (Spain) |
| 78-158 | *B. melitensis* | 1 | Isolated from a sheep (Vienne, France) |
| 88-44 | *B. melitensis* | 1 | Clinical isolate (Israel) |
| 1109 | *B. melitensis* | 1 | Clinical isolate from a 74-yr old patient travelling back from India in 2013 (Nimes, France). *B. melitensis* biovar 1 "Indian type" |
| Rev1 | *B. melitensis* | 1 | Vaccine strain (resistant to streptomycin; efficient vaccine for the prophylaxis of brucellosis in small ruminants). |
| 16M+GFP | *B. melitensis* | 1 | Strain 16M transformed with a prokaryotic expression vector encoding GFP (lab-generated fluorescent strain for research). |
| 72-59 | *B. melitensis* | 3 | Isolated from a goat (France, Dept.38) |
| 77-47 | *B. melitensis* | 3 | Clinical isolate (France, Dept. 83) |
| 78-13 | *B. melitensis* | 3 | Isolated from a goat (France, Dept.74) |
| 81-44 | *B. melitensis* | 3 |  |
| 81-140 | *B. melitensis* | 3 | Clinical isolate (France, Corsica) |
| 82-73 | *B. melitensis* | 3 | Clinical isolate (France, Dept. 31) |
| 82-87 | *B. melitensis* | 3 | Clinical isolate (Portugal) |
| 90-129 | *B. melitensis* | 3 | Isolated from a bovine (Greece) |
| 91-244 | *B. melitensis* | 3 | Isolated from a sheep (Tunisia) |
| 79-185 | *B. melitensis* | 3 | Clinical isolate (Spain) |
| CCM4915 | *B. microti* |  | *Brucella microti* type strain (BCCN 07-01). Isolated from a common vole *(Microtus arvalis)* [9] |
| 5K33 | *B. neotomae* |  | *Brucella neotomae* type strain (ATCC 23459). Isolated from a desert wood rat in the USA [10] |
| 76250 | *B. ovis* |  |  |
| 91268 | *B. ovis* |  |  |
| 91212 | *B. ovis* |  |  |
| 56/94 | *B. pinnipedialis* |  | Isolated from a hooded seal (Inverness, Scotland) [6] |
| 96/408 | *B. pinnipedialis* |  | Isolated from a harbour seal (Washington state, USA). Also named F8/99 [7] |
| UK9/99 | *B. pinnipedialis* |  | Isolated from a hooded seal (*Cystophora cristata*) in Scotland. Also know as M163/99/10 [8] |
| 04-1361Sisak-4 | *B. suis* | 1 | Isolated from a wild boar in 2004 (Croatia) |
| 15/95 | *Brucella sp.* |  | Isolated from a common seal (Inverness, Scotland). Suspected to belong to the species *B. pinnipedialis* (based on its host and CO_2_ requirement). |
| 49/94 | *Brucella sp.* |  | Isolated from a white-sided dolphin (Inverness, Scotland). Suspected to belong to the species *B. ceti* (based on its host and CO_2_ requirement). |
| NF2637 | *Brucella sp.* |  | Atypical strains isolated from wild rodents in Australia [11] |
| NF2653 | *Brucella sp.* |  | Atypical strains isolated from wild rodents in Australia [11] |
| 02/611 | *Brucella sp.* |  | Clinical strain from a 43-yr old patient with osteomyelitis in 2002 (New Zealand). Molecular analysis identified this strain as *B. ceti*-like patient with osteomyelitis. Also named F5/02 [12]. |
| B13-0095 | *Brucella sp.* |  | *B. inopinata*-like strain isolated from a PacMan frog (Texas, USA) [13] |
| ATCC48188 | *O. anthropi* |  | *Ochrobactrum anthropi* reference strain |
| LMG3301 | *O. intermedium* |  | *Ochrobactrum intermedium* type strain |

References :

1. Miller WG, Adams LG, Ficht TA, Cheville NF, Payeur JP, Harley DR, et al. *Brucella*-induced abortions and infection in bottlenose dolphins (*Tursiops truncatus*). J Zoo Wildl Med. 1999;30: 100–10.

2. De BK, Stauffer L, Koylass MS, Sharp SE, Gee JE, Helsel LO, et al. Novel *Brucella* Strain (BO1) Associated with a Prosthetic Breast Implant Infection. J Clin Microbiol. 2008;46: 43–49. doi:10.1128/JCM.01494-07

3. Tiller R V, Gee JE, Lonsway DR, Gribble S, Bell SC, Jennison A V, et al. Identification of an unusual *Brucella* strain (BO2) from a lung biopsy in a 52 year-old patient with chronic destructive pneumonia. BMC Microbiol. 2010;10: 23. doi:10.1186/1471-2180-10-23

4. Schlabritz-Loutsevitch NE, Whatmore AM, Quance CR, Koylass MS, Cummins LB, Dick Jr EJ, et al. A novel *Brucella* isolate in association with two cases of stillbirth in non-human primates - first report. J Med Primatol. 2009;38: 70–73. doi:10.1111/j.1600-0684.2008.00314.x

5. Garin-Bastuji B, Mick V, Le Carrou G, Allix S, Perrett LL, Dawson CE, et al. Examination of taxonomic uncertainties surrounding *Brucella abortus* bv. 7 by phenotypic and molecular approaches. Appl Environ Microbiol. 2014;80: 1570–9. doi:10.1128/AEM.03755-13

6. Whatmore AM, Dawson C, Muchowski J, Perrett LL, Stubberfield E, Koylass M, et al. Characterisation of North American *Brucella* isolates from marine mammals. Roop RM, editor. PLoS One. 2017;12: 1–17. doi:10.1371/journal.pone.0184758

7. Ewalt DR, Payeur JB, Martin BM, Cummins DR, Miller WG. Characteristics of a *Brucella* Species from a Bottlenose Dolphin (*Tursiops Truncatus*). J Vet Diagnostic Investig. 1994;6: 448–452. doi:10.1177/104063879400600408

8. Groussaud P, Shankster SJ, Koylass MS, Whatmore AM. Molecular typing divides marine mammal strains of *Brucella* into at least three groups with distinct host preferences. J Med Microbiol. 2007;56: 1512–1518. doi:10.1099/jmm.0.47330-0

9. Scholz HC, Hubalek Z, Sedláček I, Vergnaud G, Tomaso H, Al Dahouk S, et al. *Brucella microti* sp. nov., isolated from the common vole *Microtus arvalis*. Int J Syst Evol Microbiol. 2008;58: 375–382. doi:10.1099/ijs.0.65356-0

10. Stoenner HG, Lackman DB. A new species of *Brucella* isolated from the desert wood rat, *Neotoma lepida Thomas*. Am J Vet Res. 1957;18: 947–51.

11. Tiller R V, Gee JE, Frace MA, Taylor TK, Setubal JC, Hoffmaster AR, et al. Characterization of novel *Brucella* strains originating from wild native rodent species in North Queensland, Australia. Appl Environ Microbiol. 2010;76: 5837–5845. doi:10.1128/AEM.00620-10

12. McDonald WL, Jamaludin R, Mackereth G, Hansen M, Humphrey S, Short P, et al. Characterization of a *Brucella* sp. strain as a marine-mammal type despite isolation from a patient with spinal osteomyelitis in New Zealand. J Clin Microbiol. 2006;44: 4363–4370. doi:10.1128/JCM.00680-06

13. Soler-Lloréns PF, Quance CR, Lawhon SD, Stuber TP, Edwards JF, Ficht TA, et al. A *Brucella* spp. Isolate from a Pac-Man Frog (*Ceratophrys ornata*) Reveals Characteristics Departing from Classical Brucellae. Front Cell Infect Microbiol. 2016;6: 116. doi:10.3389/fcimb.2016.00116
