# Supplementary material for: A MALDI-TOF MS database with broad genus coverage for species-level identification of Brucella
Source: PLoS Negl Trop Dis. 2018 Oct 18;12(10):e0006874. doi: 10.1371/journal.pntd.0006874 (PMC6207331; doi:10.1371/journal.pntd.0006874)
Supplement: S3 Table — The culture conditions (media, time of incubation in hours, ± 5% CO2) are indicated. BAS = Brucella agar with 5% sheep blood, hemin & vitamin K1 (Becton Dickinson); BBA = Brucella blood agar (bioMérieux); CHOC-O = Chocolate agar plate with vitox (Oxoïd); COS-B = Columbia Blood Agar (bioMérieux, 43 041); COS-O = Columbia agar with 5% sheep blood (Oxoïd); MHB = Mueller Hinton agar with 5% sheep blood (Biorad); MHF = Mueller Hinton agar with 5% horse blood and β-NAD (Biorad); TSA-S = Trypticase soy agar with 5% sheep blood (Becton Dickinson). (DOCX) [file pntd.0006874.s004.docx]

**Table S3**

|  | **IDENTIFICATION RESULTS** | | | |
| --- | --- | --- | --- | --- |
| **Culture condition** | **# of peaks** | **Identification type** | **Identification** | **% ID** |
| BAS (48h) | 133 | Single choice | *Brucella melitensis* | 99.99 |
|  | 116 | Single choice | *Brucella melitensis* | 99.99 |
| BBA (48h) | 169 | Single choice | *Brucella melitensis* | 99.99 |
|  | 180 | Single choice | *Brucella melitensis* | 99.99 |
| BBA (96h) | 154 | Single choice | *Brucella melitensis* | 99.99 |
|  | 138 | Single choice | *Brucella melitensis* | 99.99 |
| CHOC-O (48h) | 137 | Single choice | *Brucella melitensis* | 99.90 |
|  | 117 | Single choice | *Brucella melitensis* | 99.96 |
| COS-B (24h) | 128 | Single choice | *Brucella melitensis* | 99.99 |
|  | 143 | Single choice | *Brucella melitensis* | 99.99 |
| COS-B + 5% CO_2_ (48h) | 160 | Single choice | *Brucella melitensis* | 99.98 |
|  | 154 | Single choice | *Brucella melitensis* | 99.99 |
| COS-B (96h) | 172 | Single choice | *Brucella melitensis* | 99.99 |
|  | 172 | Single choice | *Brucella melitensis* | 99.99 |
| COS-B + 5% CO_2_ (96h) | 128 | Single choice | *Brucella melitensis* | 96.56 |
|  | 183 | Single choice | *Brucella melitensis* | 99.99 |
| COS-O (48h) | 121 | Single choice | *Brucella melitensis* | 99.99 |
|  | 103 | Single choice | *Brucella melitensis* | 99.99 |
| MHB (48h) | 115 | Single choice | *Brucella melitensis* | 99.99 |
|  | 127 | Single choice | *Brucella melitensis* | 99.99 |
| MHF (48h) | 90 | Single choice | *Brucella melitensis* | 99.99 |
|  | 126 | Single choice | *Brucella melitensis* | 99.99 |
| TSA-S (48h) | 119 | Single choice | *Brucella melitensis* | 99.99 |
|  | 100 | Single choice | *Brucella melitensis* | 99.99 |
